# Evaluating the causal relationship between five modifiable factors and the risk of spinal stenosis: a multivariable Mendelian randomization analysis

**DOI:** 10.7717/peerj.15087

**Published:** 2023-03-21

**Authors:** Bangbei Wan, Ning Ma, Weiying Lu

**Affiliations:** 1 Reproductive Medical Center, Hainan Women and Children’s Medical Center, Haikou, Hainan, China; 2 Department of Urology, Haikou Affiliated Hospital of Central South University, Xiangya School of Medicine, Haikou, Hainan, China

**Keywords:** Spinal stenosis, Educational attainment, Mendelian randomization, Genome-wide association study, Causal investigation

## Abstract

**Background:**

Spinal stenosis is a neurological disorder related to the compression of the spinal cord or nerve roots, and its incidence increases yearly. We aimed to use Mendelian randomization (MR) to investigate the causal relationship between several modifiable risk factors and the risk of spinal stenosis.

**Methods:**

We obtained genome-wide association study summary data of large-sample projects (more than 100,000 individuals) from public databases. The data were associated with traits, including years of schooling (educational attainment) from the IEU OpenGWAS Project, smoking behavior (never *vs.* initiation) from the IEU OpenGWAS Project, body mass index (BMI) from the UK Biobank, length of mobile phone use from the UK Biobank, time spent watching television (TV) from the UK Biobank, and spinal stenosis from FinnGen biobank. Spinal stenosis was used as the outcome, whereas the other four traits were used as exposures. Inverse variance weighted (IVW) regressions were used as a primary to estimate the causal-effect size. Several sensitive analyses (including consistency, heterogenicity, and pleiotropy analyses) were conducted to test the stability and reliability of causal estimates.

**Results:**

Univariable MR analyses showed that genetically predicted higher educational attainment (IVW; odds ratio (OR) = 0.606; 95% confidence interval (CI): 0.507–0.724; *P* = 3.37 × 10^−8^) and never smoking (IVW; OR = 1.388; 95% CI [1.135–1.697]; *P* = 0.001) were negatively correlated with the risk of spinal stenosis. Meanwhile, a higher BMI (IVW; OR = 1.569; 95% CI [1.403–1.754]; *P* = 2.35 × 10^−8^), longer time spent using a mobile phone (IVW; OR = 1.895; 95% CI [1.306–2.750]; *P* = 0.001), and watching TV (IVW; OR = 1.776; 95% CI [1.245–2.532]; *P* = 0.002) were positively associated with the risk of spinal stenosis. Multivariable MR analysis indicated that educational attainment (IVW; OR = 0.670; 95% CI [0.465–0.967]; *P* = 0.032) and BMI (IVW; OR = 1.365; 95% CI [1.179–1.580]; *P* = 3.12 × 10^−5^) were independently causally related to the risk of spinal stenosis.

**Conclusion:**

Our findings supported the potential causal associations of the five factors (educational attainment, smoking behavior, BMI, length of mobile phone use, and watching TV) with the risk for spinal stenosis. While replication studies are essential, these findings may provide a new perspective on prevention and intervention strategies directed toward spinal stenosis.

## Introduction

Spinal stenosis is a common painful chronic disease caused by spinal-cord compression and seriously affects the quality of life of affected people (Jensen et al., 2021; Melancia, Francisco & Antunes, 2014). Spinal stenosis is divided into three types based on the narrow location, including lumbar, cervical, and thoracic spinal stenoses. The lumbar and cervical spinal stenoses are the most common types. Epidemiological investigations indicate that lumbar stenosis affects more than 103 million persons worldwide, especially older populations (Katz et al., 2022). Activity modification, analgesia, and physical therapy are the first-line treatments for spinal stenosis, but their curative effect is poor, and many patients require further therapy by surgery. Based on such harmful influence, early seeking out risk factors and implementing interventions are crucial to reducing the risk of spinal stenosis.

Spinal stenosis is an intervertebral-disc degeneration disease, and its etiologies remain unclear. Existing evidence has suggested that some bad behaviors and diseases can increase the risk for spinal stenosis (Bagley et al., 2019; Karsenty et al., 1985). The length of mobile phone use and the time spent watching television are all critical indicators for indirectly reflecting sedentary behavior and physical inactivity. Importantly, sedentary behavior and physical inactivity are detrimental to public health and can induce some the disorders, such as obesity, diabetes, and cardiovascular disease (Arocha Rodulfo, 2019; Tomkins-Lane et al., 2013). Additionally, some observational studies have reported that overweight or obesity is positively associated with the risk of spinal stenosis and may trigger factors affecting spinal stenosis (Fortin et al., 2017; Hirano et al., 2013). Furthermore, a prospective cohort study with a large sample has shown an association of smoking behavior with the risk of spinal stenosis, as well as a dose correlation (Knutsson et al., 2018). The above studies demonstrate an association between several factors and the risk of spinal stenosis; however, causal inferences are lacking. Therefore, we propose a hypothesis that these factors may have a causal relationship with the risk of spinal stenosis.

A dependable causality is advantageous and provides robust supporting evidence in drawing up and implementing public-health policies. Mendelian randomization (MR) is a newly emerging field that uses genetic variants as instrumental variables to investigate the causal relationship between exposure(s) and outcome(s) (Emdin, Khera & Kathiresan, 2017; Holmes, Ala-Korpela & Smith, 2017). In the present work, we investigated the causal relationship of several modifiable factors (years of schooling, smoking behavior (never *vs.* initiation), body mass index (BMI), length of mobile phone use, and time spent watching television (TV)) with the risk of spinal stenosis by using two-sample MR. The inverse variance weighted (IVW) algorithm was adopted as a primary computing method to assess the causal-effect size. Given the harmfulness of spinal stenosis to public health, determining causality between potential risk factors and spinal stenosis is essential to aid the establishment of prevention strategies.

## Methods and Materials

### Study design and data sources

**Figure 1:**
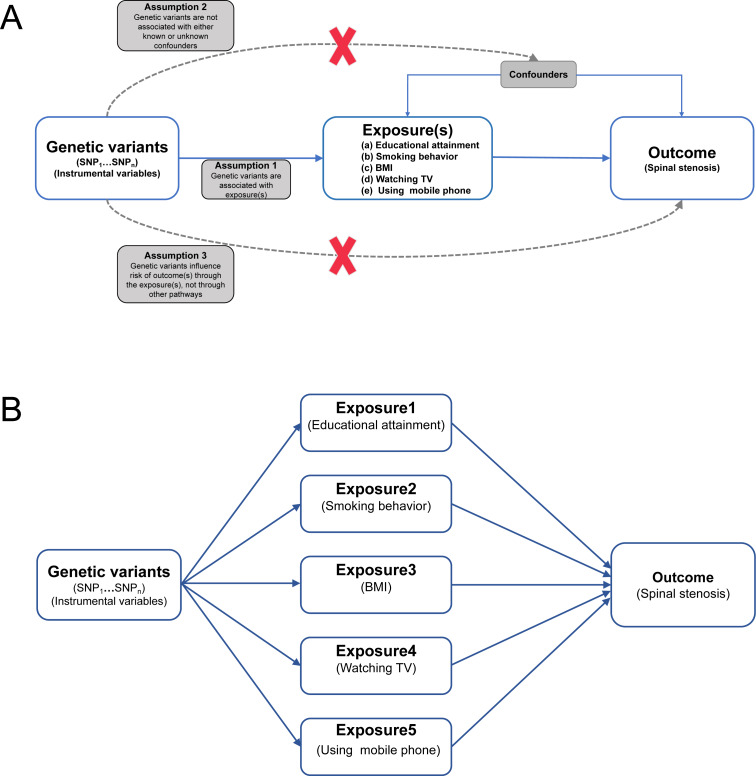
Flowchart of univariable and multivariable MR analyses. (A) Univariable MR. MR has three fundamental assumptions. (1) Relevance assumption: the genetic variants (instrumental variables) must be strongly correlated with exposure(s) (*P* < 5 × 10^−8^) (*r*^2^ < 0.001 and distance > 10 000 kb, the SNPs in pairwise linkage disequilibrium). (2) Independence assumption: no unmeasured confounders of the correlations existed between genetic variants and outcome(s). (3) Exclusion restriction assumption: the genetic variants influenced the outcome(s) only *via* exposure(s). (B) Multivariable MR: MR, Mendelian randomization; educational attainment, years of schooling; BMI, body mass index; smoking behavior, smoking behavior (never *vs.* initiation); using mobile phone, length of mobile phone use; watching TV, time spent watching television; and SNP, single nucleotide polymorphism.

The three critical basic assumptions and overall study design flowchart of the MR are displayed in Fig. 1. In this work, the exposures- and outcome-related genome-wide association study (GWAS) summary data of European populations were extracted from an available public database (the IEU Open GWAS database: https://gwas.mrcieu.ac.uk/) and used to perform MR analysis. The data details were as follows. Educational attainment (years of schooling, standard deviation (SD): 4.2 years)-related summary data with a GWAS sample size of 766 345 individuals were derived from the IEU OpenGWAS Project (Lee et al., 2018). BMI (SD: 4.75 kg/m^2^)-related genetic data were from a GWAS meta-analysis involving 681 275 individuals and extracted from the UK Biobank (Yengo et al., 2018). Smoking behavior (never *vs.* initiation)-related statistical data were from a GWAS meta-analysis with 311 629 cases and 321 173 controls and extracted from the IEU OpenGWAS Project (Liu et al., 2019). Using mobile (length of mobile phone use)-related summary-level data of 456 972 volunteers were derived from a GWAS analysis of the UK Biobank. Watching TV (time spent watching TV; SD: 1.62 h/day)-related summary genetic data of 437 887 individuals were also from a GWAS analysis of the UK Biobank. The spinal stenosis GWAS summary statistical data, which involved 9,169 spinal stenosis cases and 164 682 controls, were extracted from the FinnGen research project.

### Statistical analysis

#### Univariable and multivariable MR analyses

Univariable inverse variance weighted (IVW) regression was used as a principal algorithm to estimate causal-effect size from exposure(s) to outcome. We identified single-nucleotide polymorphisms (SNPs) independently correlated with exposures (educational attainment, smoking behavior, BMI, using mobile, and watching TV) and used them as instrumental variables to assess the causal relationship between exposure and outcome. The independent SNPs were selected according to the following parameters: (a) *P* < 5.0 × 10^−8^ was regarded as a statistically significant threshold for a strong correlation between SNPs and exposure; and (b) *r*^2^ < 0.001 and distance >10,000 kb among SNPs in pairwise linkage disequilibrium (LD) were deemed the independent threshold. The F statistic was utilized to estimate the instrument strength and its computing method, as described in a previous study (Pierce, Ahsan & Vanderweele, 2011). An F statistic >10 was considered as having no weak instrument bias.

Next, we conducted a series of sensitivity analyses to validate the robustness and reliability of the univariate MR analyses. The MR Steiger test was used to inspect the correctness of causal hypotheses in the MR analyses. Four methods including the MR–Egger (Bowden, Davey Smith & Burgess, 2015), maximum likelihood (Xue, Shen & Pan, 2021), MR–pleiotropy residual sum outlier (MR-PRESSO) (Verbanck et al., 2018), and robust adjusted profile score (MR-RAPS) (Zhao et al., 2020) were adopted to prove the consistency of causal hypothesis in IVW analysis. The statistical power of univariable MR analyses was computed using an available online tool (https://shiny.cnsgenomics.com/mRnd/) (Brion, Shakhbazov & Visscher, 2013). A power greater than 80% was deemed as excellent statistical evidence. Cochran’s Q statistics in the IVW and MR–Egger models were used to assess the heterogeneity of SNPs. *P* < 0.05 was deemed to indicate significant heterogeneity. The MR–PRESSO, MR–Egger, and IVW approaches were utilized to identify and remove potential outliers that can cause underlying pleiotropy. MR–Egger regression was used to determine whether a potential pleiotropy existed in univariable MR analysis. The leave-one-out permutation method was used to examine whether an existing single SNP can alter the pooled effect of all SNPs in IVW analysis. The MR Steiger test was utilized to determine whether the causal assumption was correct.

Finally, considering the importance of the five factors for the risk of spinal stenosis, we further included the five factors to conduct a multivariable MR analysis (Lu, Wan & Sun, 2022) to identify the independent exposure(s).

All statistical analyses of MR were conducted using the TwoSampleMR (version 0.5.6) (Hemani, Tilling & Davey Smith, 2017) and MRPRESSO (version 1.0) (Verbanck et al., 2018) packages in R software (version 4.1.2; R Core Team, 2021).

## Results

### Univariable and multivariable MR analysis

**Figure 2:**
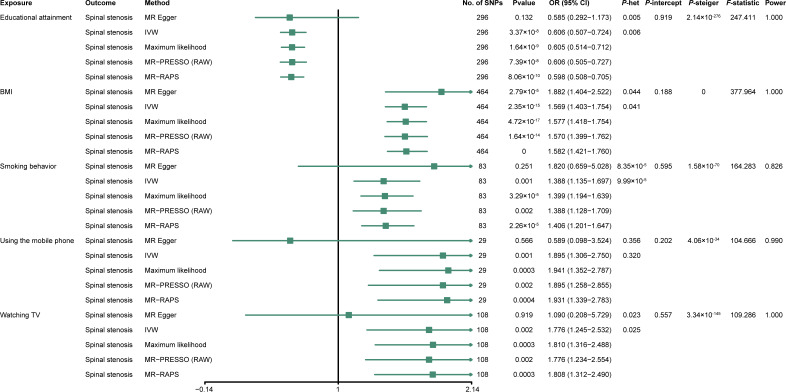
Forest plot showing univariable MR analysis. OR, odds ratio; CI, confidence interval; *P*-het, *P* value for heterogeneity using Cochran Q test; *P*-intercept, *P* value for MR-Egger intercept; *P*-Steiger, *P* value for MR–Steiger test; IVW, inverse variance weighted; MR–PRESSO, Mendelian randomization–pleiotropy residual sum outlier; MR–RAPS, robust adjusted profile score; SNP, single-nucleotide polymorphism; educational attainment, years of schooling; BMI, body mass index; smoking behavior, smoking behavior (never *vs.* initiation); using mobile phone, length of mobile phone use; and watching TV, time spent watching television.

First, to clarify the potential causal relationship between each exposure (educational attainment, smoking behavior, BMI, mobile phone use, and watching TV) and outcome (spinal stenosis), we performed univariable MR analyses using IVW regression. The results from univariable MR analyses showed that a 1-SD increase in years of schooling was correlated with a 39.40% reduction in the spinal stenosis risk (IVW; odds ratio (OR) = 0.606, 95% confidence interval (CI) [0.507–0.724]; *P* = 3.37 ×10^−8^) (Fig. 2); Smoking was associated with a 38.30% increase in the spinal stenosis risk (IVW; OR = 1.388; 95% CI [1.135–1.697]; *P* = 0.001) (Fig. 2); A 1-SD increase in BMI was correlated with a 56.90% rise in the risk of spinal stenosis (IVW; OR = 1.569; 95% CI [1.403–1.754]; *P* = 2.35 ×10^−15^) (Fig. 2); Longer time spent watching TV (IVW; OR = 1.776; 95% CI [1.245–2.532]; *P* = 0.002) and using mobile phone (IVW; OR = 1.895; 95% CI [1.306–2.750]; *P* = 0.001) were significantly associated with 77.60% and 89.50% increase in the risk of spinal stenosis, respectively (Fig. 2). Results of univariable MR analyses showed no potential weak-instrument bias (all *F* statistics >10). Moreover, the power value of each analysis was almost 100%, indicating outstanding reliability (Fig. 2). All included SNPs are exhibited in Tables S1–S5.

**Figure 3:**
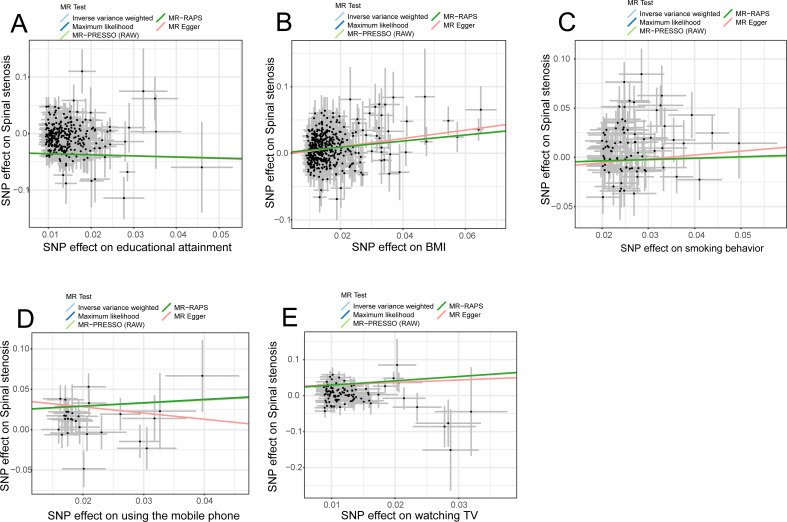
Scatter plots indicated the IVW regression direction tested by four methods. MR, Mendelian randomization; SNP, single-nucleotide polymorphism; IVW, inverse variance weighted; MR–PRESSO, Mendelian randomization–pleiotropy residual sum outlier; MR–RAPS, robust adjusted profile score; educational attainment, years of schooling; BMI, Body mass index; smoking behavior, smoking behavior (never *vs.* initiation); using mobile phone, length of mobile phone use; watching TV, time spent watching television. (Figure created by Zhi Zhou).

Sensitivity analyses were subsequently conducted to examine the stability and dependability of the univariable studies. Results from the four methods (MR–Egger, maximum likelihood, MR–PRESSO, and MR-RAPS) were almost similar to the IVW estimates (Fig. 3). Results of heterogeneity analyses indicated certain heterogeneities among the four univariable analyses (educational attainment, smoking behavior, BMI, and time spent watching TV) (Fig. 2). The heterogeneities may have originated from Mendel’s law of independent assortment rather than existing pleiotropy (Lewis & Simpson, 2022; Qi, 2009). The MR–Egger regressions indicated no unbalanced horizontal pleiotropy in the MR analyses (all *P*_−*intercept*_ >0.05) (Fig. 2). The iterative leave-one-out test displayed no single SNP that influenced the univariable results (all *P* < 0.01) (Tables S6–S10). Results of the MR Steiger test showed that all causal assumptions were correct (all *P* < 0.01) (Fig. 2).

**Figure 4:**
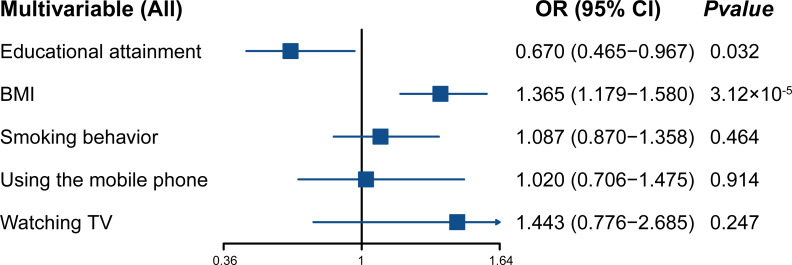
Forest plot displaying multivariable MR analysis. OR, odds ratio; CI, confidence intervals; educational attainment, years of schooling; BMI body mass index; smoking behavior, smoking behavior (never *vs.* initiation); using mobile phone, length of mobile phone use; watching TV, time spent watching television.

Finally, considering the importance of the five factors for the risk of spinal stenosis, we also conducted multivariable MR analysis to reduce the effect of confoundings and identify the independent exposure(s). We observed that educational attainment and BMI were independently associated with the risk of spinal stenosis in multivariable MR (Fig. 4). Results suggested a direct causal relationship between the two factors (educational attainment and BMI) and the risk of spinal stenosis.

## Discussion

We used GWAS summary-level data from large-sample studies to investigate the causal link between the five factors (educational attainment, smoking behavior, BMI, length of mobile phone use, and time spent watching TV) and the risk of spinal stenosis. We observed a causal association between the five factors and the risk of spinal stenosis; educational attainment and BMI were independently correlated with the risk of spinal stenosis. The three factors (smoking behavior, length of mobile phone use, and time spent watching TV) may impact the risk of spinal stenosis by regulating other factors, such as obesity and degenerative changes in the spine.

Spinal stenosis is characterized by chronic back pain and occurs in aging populations (Austevoll et al., 2021). The people affected by spinal stenosis are gradually becoming younger, thereby seriously affecting their quality of life (Katz et al., 2022; Lurie & Tomkins-Lane, 2016). Therefore, ascertaining risk factors and mapping out effective public strategies to prevent spinal stenosis is necessary and urgent. Poor lifestyle behaviors are apparent risk factors for spinal stenosis, whereas changes in the underlying molecular mechanism are intrinsic pathogeny for spinal stenosis (Byvaltsev et al., 2022). Previous studies have reported that a higher educational level is a protective factor for many diseases, such as type 2 diabetes (Agardh et al., 2011), coronary heart disease (Falkstedt & Hemmingsson, 2011), osteoporosis (Ho, Chen & Woo, 2005), etc. Although a recent study has also shown a spinal-stenosis risk difference in the populations with different educational levels, the difference vanishes if controlling for other confoundings (age, gender, and BMI) (Yabuki et al., 2013). Similarly, our work indicated that educational attainment was negatively correlated with the risk of spinal stenosis in univariable MR analysis. Interestingly, when adjusting for the other four factors, educational attainment still independently influences the risk of spinal stenosis. Statistical evidence of several sensitivity analyses also strongly supported our findings. Based on the above findings, increasing educational attainment may be a very beneficial strategy to attenuate the risk of spinal stenosis.

Smoking behavior and BMI were risk factors for spinal stenosis (Bagley et al., 2019). A recent study involving 331 941 individuals revealed that individuals with smoking behavior have a higher risk of spinal stenosis, and the risk increases with increased smoking dose (Knutsson et al., 2018). Likewise, our result supported a causal association between smoking and the risk of spinal stenosis. Smoking behavior increased the risk for spinal stenosis by 38.80% in univariable MR analysis. Still, multivariable MR analysis showed that smoking was not an independent risk factor for spinal stenosis after correcting four factors (educational attainment, BMI, length of mobile phone use, and time spent watching TV). These results suggested that smoking could impact the risk of spinal stenosis by mediating other factors. BMI is also one of the risk factors for spinal stenosis. In a previous cohort study including 364 467 individuals, a higher BMI is found to be associated with a higher risk of spinal stenosis (Knutsson et al., 2015). In our work, the causal inference of univariable investigation showed that increased BMI was associated with a high risk of spinal stenosis. Additionally, the multivariable MR analysis result denoted a direct causal relationship between BMI and the risk of spinal stenosis. Collectively, these findings preliminarily indicated that losing weight and stopping smoking may be very helpful in preventing spinal stenosis.

Using a mobile phone or watching TV for a long time is terrible behavior and closely related to public health (Foreman et al., 2021; Ghavamzadeh, Khalkhali & Alizadeh, 2013; Henschel, Gorczyca & Chomistek, 2020; Ikinci Keles & Uzun Sahin, 2021; Madhav, Sherchand & Sherchan, 2017). No literature has reported the relationship of using a mobile phone or watching TV for a long time with the risk of spinal stenosis. In the present work, we revealed for the first time an indirect causal relationship between the two modifiable factors (using a mobile phone and watching TV for a long time) and the risk of spinal stenosis.

Our work has some strengths and limitations. One of the strengths was our use of GWAS summary-level data from recent extensive sample studies, and the included SNPs were more comprehensive. Another strength was our use of several sensitivity analyses including heterogeneity tests, pleiotropy, and robustness assessment to improve the results’ reliability and stability. Last but not least, our work makes up for the observational study deficiency that lacks causal inference. Existing limitations are also inevitable in our work. First, we used the GWAS data derived from European ancestry populations; whether the findings can ultimately be generalized to non-European ancestry populations remains unclear. Second, although our work primarily revealed the causal relationship of five modifiable risk factors with the risk of spinal stenosis, the causal effect’s underlying mechanisms remain unexplained. Third, data limitations mean we cannot achieve a stratified analysis for parameters such as gender and age.

In conclusion, we used the extensive GWAS summary data to investigate the association of five factors (educational attainment, smoking behavior, BMI, length of mobile phone use, and time spent watching TV) with the risk of spinal stenosis *via* MR methods. We found that when correcting other confoundings, a direct causal relationship existed between two factors (educational attainment and BMI) and the risk of spinal stenosis, whereas an indirect causal correlation existed between the other three factors (smoking behavior, length of mobile phone use, and time spent watching TV) and the risk of spinal stenosis. These findings provided preliminary evidence to support the fact that elevating educational attainment and reducing BMI can help attenuate the risk of spinal stenosis. Moreover, we need to explore possible mediators before implementing interventions for these three factors (smoking behavior, length of mobile phone use, and time spent watching TV) to reduce the risk of spinal stenosis. Our findings provided insights into drawing up public policies to prevent spinal stenosis.

##  Supplemental Information

10.7717/peerj.15087/supp-1 Supplemental Information 1 Raw data, the independent SNPs’ details, and leave-one-out analyses’ results

10.7717/peerj.15087/supp-2 Supplemental Information 2 The STROBE-MR checklist and R Code

## Additional Information and Declarations

### Data Availability

The following information was supplied regarding data availability: The raw measurements, the STROBE-MR checklist, RAW data and R code are available in the Supplementary Files.
